# An Atypical Case of Extrapulmonary Sarcoidosis with Severe Hypercalcemia as Initial Presentation, Successfully Treated with Glucocorticoids

**DOI:** 10.3390/clinpract14040102

**Published:** 2024-06-29

**Authors:** Sushmita Mittal, Karolina Pogorzelski, Christopher Huxel, Chokkalingam Siva, Deepthi Rao

**Affiliations:** 1 Department of Medicine, University of Missouri, Columbia, MO 65212, USA; huxelc@health.missouri.edu; 2 School of Medicine, University of Missouri, Columbia, MO 65212, USA; kpmk2@umsystem.edu; 3 Department of Rheumatology, University of Missouri, Columbia, MO 65212, USA; 4 Department of Pathology, University of Missouri, Columbia, MO 65212, USA; raods@health.missouri.edu

**Keywords:** sarcoidosis, hypercalcemia, glucocorticoids

## Abstract

Background: Sarcoidosis is a multisystemic disease that is histologically characterized by non-caseating granulomas in one or more organs. Although hypercalcemia is commonly seen in sarcoidosis, clinically significant hypercalcemia as the initial presentation of sarcoidosis is exceedingly rare. Long-standing hypercalcemia can lead to several complications and needs to be adequately managed to prevent irreversible damage. Currently, there are no standard treatment guidelines for sarcoidosis-induced hypercalcemia, although glucocorticoids have often been used as first-line therapy. Case Report: We describe a 55-year-old male patient who presented with dull right upper quadrant abdominal pain and a 30-pound weight loss over one month. He was found to have severe hypercalcemia, which was treated with intravenous (IV) normal saline and intramuscular calcitonin. Imaging studies revealed hypodense lesions throughout the bilateral hepatic lobes, spleen, and bilateral kidneys, with no pathologic mediastinal, hilar, supraclavicular, or axillary lymphadenopathy or pulmonary parenchymal disease. A splenic biopsy confirmed extrapulmonary sarcoidosis. After initial discharge, the patient was re-admitted weeks later for severe hypercalcemia, which was successfully treated with the initiation of prednisone. Conclusions: In this report, we present an atypical case of isolated extrapulmonary sarcoidosis with severe hypercalcemia as the initial presentation, successfully treated with steroids.

## 1. Introduction

Sarcoidosis is an inflammatory condition of unknown etiology, where non-caseating granulomas form in one or more systems in the body. Sarcoidosis can also present with hypercalcemia in about 7–18% of cases, though patients rarely present with severe hypercalcemia as the first presentation, as seen in our patient [1].

Sarcoidosis causes a parathyroid hormone (PTH)-independent hypercalcemia due to increased 1-alpha hydroxylase activity in macrophages that form part of the granuloma [2]. This enzyme increases the conversion of inactive 25-hydroxyvitamin D into active 1,25-dihydroxyvitamin D in the kidneys without any type of homeostatic control. This increase in active vitamin D promotes calcium absorption from the intestines and suppresses PTH, leading to increased serum calcium levels [3,4].

Although there are no standard guidelines for the treatment of hypercalcemia in sarcoidosis, corticosteroids are often used as the first-line treatment. Patients who do not respond to steroid treatment within 2 weeks are thought to have other causes of hypercalcemia. Several studies also discuss the benefits of using chloroquine, ketoconazole, and infliximab for the treatment of sarcoidosis-induced hypercalcemia, although these are rarely used [1]. In our paper, we report a rare case of severe hypercalcemia seen in extrapulmonary sarcoidosis that improved with prednisone.

## 2. Case Report

We present a case of a 55-year-old Caucasian male with a past medical history of prediabetes and erectile dysfunction who presented to the Emergency Department with dull right upper quadrant (RUQ) abdominal pain for one month. The abdominal pain was insidious in onset and associated with intermittent nausea and vomiting. He had an inability to tolerate both solids and liquids, resulting in a 30-pound weight loss. He denied any sick contacts, shortness of breath, hemoptysis, or chest pain. Family and social history were insignificant. The patient was not taking any medications prior to his admission to the hospital.

On presentation, he was afebrile with a blood pressure of 124/83 mmHg, a heart rate of 92 beats per minute, and an oxygen saturation of 99% on room air. His physical examination revealed tenderness upon palpation in the RUQ. The initial laboratory workup revealed a severely elevated serum calcium (14.3 mg/dL) and ionized calcium (1.46 mmol/L), which required immediate treatment with intravenous (IV) normal saline and intramuscular calcitonin. Subsequent laboratory testing for hypercalcemia revealed a low serum parathyroid hormone (PTH) level (7.2 pg/mL), a normal parathyroid hormone related peptide level (0.6 pg/mL), a low 25-hydroxyvitamin D level (21.80 ng/mL), an elevated 1,25-dihydroxyvitamin D3 (69 pg/mL), and elevated angiotensin-converting enzyme (141 units/L) (Table 1). 

He also presented with abnormalities in his kidney and liver function tests. His kidney function tests revealed elevated blood urea nitrogen (23 mg/dL) and creatinine levels (1.69 mg/dL), with a decreased glomerular filtration rate (47 mL/min/1.73m^2^). A urinalysis, urine electrolytes, and a bladder scan were performed to further evaluate the etiology of his renal dysfunction; however, there were no abnormalities detected. His liver function tests revealed an elevated alkaline phosphatase (566 units/L) and total bilirubin (1.94 mg/dL) with a normal range of alanine aminotransferase and aspartate aminotransferase (37 units/L and 32 units/L, respectively) (Table 1). Levels of ceruloplasmin, alpha-1-antitrypsin, smooth muscle antibody, ammonia, and a hepatitis panel were all within normal limits.

Further workup with imaging was then performed to determine the cause of his renal and hepatic dysfunction. A computed tomography (CT) abdomen and magnetic resonance cholangiopancreatography (MRCP) revealed innumerable ill-defined and predominantly sub-centimeter hypodense lesions throughout the bilateral hepatic lobes, spleen, and bilateral kidneys that were too small to characterize. His imaging also revealed a 1 mm non-obstructing stone within the right ureterovesical junction. The chest CT revealed a few scattered 3–4 mm pulmonary nodules with no pathologic mediastinal, hilar, supraclavicular, or axillary adenopathy. 

**Figure 1:**
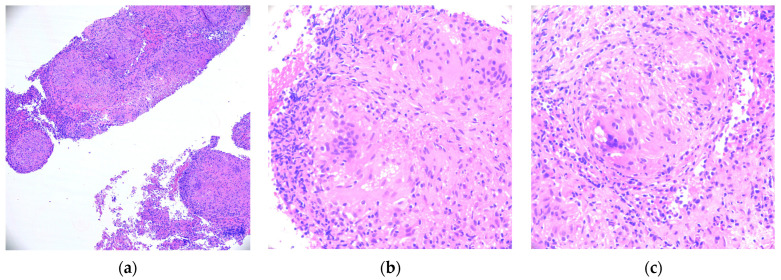
(**a**–**c**): Histopathological analysis of the case. All three subfigures show the Hematoxylin–Eosin (HE-400X) stain of a spleen biopsy specimen with numerous granulomas.

The presentation of abdominal pain, hypercalcemia, a 30-pound weight loss, and numerous lesions throughout the bilateral hepatic lobes, spleen, and bilateral kidneys initially raised concern for malignancy. A diagnostic biopsy of the spleen was performed to rule out malignancy; however, it revealed dense, non-caseating granulomatous infiltrate with negative staining for fungi or mycobacterium (Figure 1). Further testing for other etiologies of infiltrative disease all came back negative, including mycobacterium tuberculosis, histoplasmosis, hepatitis, and primary biliary cirrhosis. Due to the histopathological findings and hypercalcemia, our patient was ultimately diagnosed with extrapulmonary sarcoidosis. 

Our patient was discharged once his symptoms and calcium levels improved; however, he was re-admitted 2 weeks later for episodes of vomiting and intermittent RUQ pain. The laboratory work-up revealed elevated calcium (12.1 mg/dL), bilirubin (3.22 mg/dL), and alkaline phosphatase levels (733 units/L), compared to the previous values on discharge. The patient was started on IV fluids, and Rheumatology was also consulted for further management of sarcoidosis. He was started on a prednisone taper of 40 mg daily with a weekly reduction by 10 mg and was maintained on prednisone 5 mg daily after discharge for 7 months. He was also started on mycophenolate mofetil 500 mg twice a day for systemic sarcoidosis. Although methotrexate is the more commonly used corticosteroid-sparing therapies for sarcoidosis, we elected against using it due to his renal dysfunction. 

After discharge, our patient also established care with Nephrology for his elevated creatinine levels and was found to have chronic kidney disease (CKD) stage 3b, which was thought to be secondary to his sarcoidosis and hypercalcemia. After 2 months of steroids, our patient had marked improvement in both his serum calcium (12.1 mg/dL to 9.8 mg/dL) and 1,25 dihydroxyvitamin D levels (69 pg/mL to 14 pg/mL). He also underwent an MRCP five months after prednisone and mycophenolate mofetil initiation and was found to have significant interval improvement in splenic, hepatic, and renal lesions compared to his previous imaging. After 7 months, our patient completed his course of prednisone with stabilization of both his calcium levels and his glomerular filtration rate. 

## 3. Discussion

Sarcoidosis is a multi-system granulomatous disease that predominantly affects African-American females, typically peaking between the ages of 20 and 30. Although hypercalcemia is a well-known electrolyte abnormality seen in sarcoidosis, severe hypercalcemia of more than 14 mg/dL is rarely seen. One study performed by Baughman et al. examined 736 patients with sarcoidosis and found that only 3.7% were found to have hypercalcemia. Furthermore, these patients were predominantly Caucasian males, like our patient [5]. 

Diagnosis of sarcoidosis in the setting of hypercalcemia is challenging, as the presentation can mimic malignancy and other granulomatous diseases. Our case was even more challenging as our patient did not present with the classic pulmonary findings often seen in sarcoidosis. Hypercalcemia due to sarcoidosis typically presents with low PTH levels, low 25-hydroxyvitamin D levels, and elevated 1,25-dihydroxyvitamin D levels, which were all seen in our patient [3,4]. Our patient also tested negative for anti-mitochondrial antibodies, anti-smooth-muscle antibodies, hepatitis panel, and tuberculosis, which eliminated several differential diagnoses before a biopsy was performed. Initially, there was concern for malignancy-induced hypercalcemia in our patient with his 30-pound weight loss; however, his laboratory values revealed normal PTHrP, which decreased the likelihood of malignancy.

Renal involvement in sarcoidosis is rare and not well understood. One study by Baughman et al. revealed that out of 736 patients with sarcoidosis, only 0.7% were found to have renal involvement [5]. Currently, the most frequent causes of acute kidney injury (AKI) in sarcoidosis include hypercalcemia, interstitial nephritis with or without granulomas, or a combination of both [6]. Studies suggest that calcium abnormalities, including hypercalcemia, hypercalciuria, nephrocalcinosis, and nephrolithiasis are predominantly responsible for renal injury in sarcoidosis rather than granulomatous infiltration [7,8]. One study performed by Lebacq found that among 152 patients with sarcoidosis, impairment of renal function was most prominent in those with hypercalcemia [9]. 

On admission, our patient presented with an elevated creatinine level and glomerular filtration rate (GFR) and was eventually diagnosed with chronic kidney disease (CKD) stage 3b. While the exact cause of his kidney injury was unclear, it was likely multifactorial. We suspect a component of his kidney injury was due to severe hypercalcemia, as both the GFR and creatinine levels improved once his hypercalcemia levels were treated. Our patient also presented with nephrolithiasis with several small non-obstructing stones, which could have also contributed to his AKI. It is uncertain whether our patient had a component of sarcoidosis-induced interstitial nephritis with granuloma formation, as he was found to have several sub-centimeter hypodense lesions in his kidneys similar to the confirmed granulomas seen in his spleen. Unfortunately, the only way to confirm this is via biopsy, which was deferred in our case due to the risk outweighing the benefit. 

Management of hypercalcemia in sarcoidosis is important, as chronic hypercalcemia can lead to several long-term complications, such as renal failure and nephrolithiasis, as seen in our patient. Treatment for sarcoidosis-related hypercalcemia is often focused on decreasing 1,25-dihydroxyvitamin D levels. In fact, one study performed by Kavathia et al. demonstrated that elevated levels of 1,25-dihydroxyvitamin D are associated with prolonged immunosuppressive therapy requirements in sarcoidosis [10]. Currently, glucocorticoids remain the mainstay treatment for sarcoidosis-related hypercalcemia in either severe (>12 mg/dL) or symptomatic cases. Steroids treat hypercalcemia via several mechanisms, including the inhibition of macrophage 1-hydroxylase activity in granulomas, which decreases the formation of active 1,25 dihydroxyvitamin D [11]. 

While there are no standard guidelines for prednisone dosing in sarcoid-induced hypercalcemia, several studies suggest a starting dose of 20 to 40 mg once daily, which is often tapered to the lowest effective dose. The duration of the steroid taper also seems to vary based on the patient’s response. Variable steroid tapering regimes, ranging from 1 month to 1 year, have been described in several case studies regarding steroid-induced hypercalcemia [12,13,14]. Our patient was started on prednisone 40 mg daily with a weekly reduction by 10 mg and eventual maintenance on prednisone 5 mg for 7 months. Within 2 months of steroid initiation, our patient’s calcium and 1,25-dihydroxyvitamin D levels drastically decreased with the eventual stabilization of his GFR. This rapid response to steroids has also been seen in other case reports by Bia et al., Zhang et al., and Bell et al. [2,15,16]. 

Currently, vitamin D and calcium supplementation in patients with sarcoidosis-induced hypercalcemia remains controversial. While some suggest supplementation can lead to a higher risk of calcium-induced complications in sarcoidosis, others say it can prevent osteoporosis in patients on steroid treatment for sarcoidosis. One case series by Sodhi et al. studied 196 patients with sarcoidosis and found that the incidence of hypercalcemia was higher in the group that received vitamin D (42.3%) compared to the non-supplemented group (18.3%) [17]. This contrasts with another case series performed by Kamphuis et al., which determined that out of 104 patients who received calcium and vitamin D supplementation, only 5 patients were found to have hypercalcemia [18]. Our patient was initially advised to limit sun exposure and calcium intake; however, months later, he was found to have vitamin D deficiency (25-hydroxycholecalciferol level of 19.80 ng/mL and PTH level of 72.9 pg/mL). Given that our patient was on long term steroids that could increase his risk of osteoporosis, he was advised to start a low-dose vitamin D supplement (around 400–800 IU/day) and to avoid strictly limiting the calcium intake. Our patient’s serum calcium levels continued to remain stable despite supplement use for months. It is important for researchers to perform large cohort studies regarding the impact of vitamin D supplementation in sarcoidosis patients to increase the generalizability of the results. It is also crucial for health-care providers to monitor a patient’s calcium levels before and after taking vitamin D supplements to prevent any hypercalcemia-related complications.

## 4. Conclusions

Our case report highlights an unusual presentation of extrapulmonary sarcoidosis with symptomatic hypercalcemia as the first presentation. Chronic hypercalcemia in patients with sarcoidosis can lead to several downstream complications, including nephrolithiasis and kidney failure, as seen in our patient. Sarcoidosis-related hypercalcemia lacks well-established treatment guidelines; so, we use this case to demonstrate another case of successfully treating hypercalcemia in sarcoidosis with glucocorticoids. 

Our study is limited in that it only follows a single patient’s case; however, we use this case to support the several other case studies that present with similar findings. These cases serve to highlight the importance of performing large sample size studies to determine the overall benefits of using glucocorticoids in sarcoidosis-induced hypercalcemia.

## Figures and Tables

**Table 1:** Summary of the pertinent initial laboratory findings of the patient.

Laboratory Test	Value	Reference
White Blood Cell Count (10^9^/L)	7.62	3.5–10.5
Hemoglobin (g/L)	12.4	12.0–15.5
Platelet Count (10^9^/L)	242	150–450
Serum Calcium (mg/dL)	14.3	0.7–1.2
Ionized Calcium (mmol/L)	1.46	1.12–1.30
Creatinine (mg/dL)	1.69	0.5–1.0
Blood Urea Nitrogen (mg/dL)	23	6–20
Glomerular Filtration Rate (mL/min/1.73m^2^)	47	>90
Alkaline Phosphatase (units/L)	566	40–129
Total Bilirubin (mg/dL)	1.94	0–1.6
Aspartate Transaminase (units/L)	32	8–33
Alanine Transaminase (units/L)	37	10–35
Serum Parathyroid Hormone (pg/mL)	7.2	15–65
Parathyroid Hormone Related Peptide (pg/mL)	0.6	≤4.2
25-Hydroxyvitamin D (ng/mL)	21.80	30–80
1,25-Dihydroxyvitamin D3 (pg/mL)	69	18–64
Angiotensin-converting Enzyme (units/L)	141	16–85

## Data Availability

The datasets used and/or analyzed during the current study are available from the corresponding author on reasonable request.
